# Supplementary material for: Mechanisms governing the pioneering and redistribution capabilities of the non-classical pioneer PU.1
Source: Nat Commun. 2020 Jan 21;11:402. doi: 10.1038/s41467-019-13960-2 (PMC6972792; doi:10.1038/s41467-019-13960-2)
Supplement: Supplementary file 7 — Source data [file 41467_2019_13960_MOESM7_ESM.zip › Source_Data/FigureS2/FigureS2B_MotifScanOutput/KmeansCluster7/homerResults/motif7.similar.html]

motif7

## Information for motif7

G
T
A
C
C
T
G
A
A
T
G
C
C
G
A
T
A
C
G
T
A
G
T
C
A
C
G
T
A
T
C
G
A
G
T
C
G
C
A
T
G
C
A
T
G
A
C
T
G
C
A
T
A
G
C
T
  
Reverse Opposite:  

T
C
G
A
C
G
T
A
C
T
G
A
C
G
T
A
C
G
T
A
T
C
A
G
A
T
G
C
C
G
T
A
A
C
T
G
G
T
C
A
C
G
T
A
T
A
C
G
G
A
C
T
C
A
T
G
  

|  |  |
| --- | --- |
| p-value: | 1e-170 |
| log p-value: | -3.922e+02 |
| Information Content per bp: | 1.641 |
| Number of Target Sequences with motif | 479.0 |
| Percentage of Target Sequences with motif | 14.92% |
| Number of Background Sequences with motif | 1205.3 |
| Percentage of Background Sequences with motif | 2.76% |
| Average Position of motif in Targets | 102.1 +/- 41.9bp |
| Average Position of motif in Background | 100.6 +/- 71.1bp |
| Strand Bias (log2 ratio + to - strand density) | 0.1 |
| Multiplicity (# of sites on avg that occur together) | 1.10 |
| Motif File: | file (matrix) reverse opposite |

### Similar de novo motifs found

|  |  |  |  |  |  |  |  |
| --- | --- | --- | --- | --- | --- | --- | --- |
| Rank | Match Score | Redundant Motif | P-value | log P-value | % of Targets | % of Background | Motif file |
| 1 | 0.893 | C G T A T C A G A T G C C G T A A T C G T G C A G C T A T A C G C A G T | 1e-131 | -302.569865 | 16.64% | 4.50% | motif file (matrix) |
| 2 | 0.892 | A G T C A G T C T C G A T A G C G C A T A C G T G A T C C G A T A T C G G A T C G A C T | 1e-123 | -284.523537 | 19.66% | 6.44% | motif file (matrix) |
| 3 | 0.982 | G A T C C T G A A T G C G C A T A C G T A T G C A G C T T C A G A G T C G C A T G C A T A G C T G A C T | 1e-122 | -282.409499 | 10.59% | 1.89% | motif file (matrix) |
| 4 | 0.903 | G T C A G C T A C T A G A G T C C G T A C A T G G T C A C G T A A T C G G A C T | 1e-98 | -226.770444 | 13.83% | 4.04% | motif file (matrix) |
| 5 | 0.848 | C G T A T A C G G T A C C G T A T A C G G T C A C T G A A T C G | 1e-69 | -160.638905 | 33.30% | 19.47% | motif file (matrix) |
